# Guideline on management of suspected adverse reactions to ingested histamine: Guideline of the German Society for Allergology and Clinical Immunology (DGAKI), the Society for Pediatric Allergology and Environmental Medicine (GPA), the Medical Association of German Allergologists (AeDA) as well as the Swiss Society for Allergology and Immunology (SGAI) and the Austrian Society for Allergology and Immunology (ÖGAI)

**DOI:** 10.5414/ALX02269E

**Published:** 2021-10-05

**Authors:** Imke Reese, Barbara Ballmer-Weber, Kirsten Beyer, Sabine Dölle-Bierke, Jörg Kleine-Tebbe, Ludger Klimek, Sonja Lämmel, Ute Lepp, Joachim Saloga, Christiane Schäfer, Zsolt Szepfalusi, Regina Treudler, Thomas Werfel, Torsten Zuberbier, Margitta Worm

**Affiliations:** 1 Nutrition Therapy, Munich, Germany,; 2 Clinic for Dermatology and Allergology, Cantonal Hospital St. Gallen and Department of Dermatology, University Hospital Zurich, Switzerland,; 3 Clinic for Pediatrics with focus on Pneumology and Immunology, Charité-Universitätsmedizin – Campus Virchow-Klinikum, Berlin,; 4 Allergology and Immunology, Department of Dermatology, Venereology and Allergology, Charité-Universitätsmedizin Berlin,; 5 Allergy and Asthma Center Westend, Berlin,; 6 Center for Rhinology and Allergology, Wiesbaden,; 7 German Allergy and Asthma Association (DAAB), Mönchengladbach,; 8 Practice for Pulmonary Medicine and Allergology, Stade,; 9 Department of Dermatology, University Medical Center, Johannes Gutenberg University, Mainz,; 10 Nutrition Therapy, Schwarzenbek,; 11 Department of Pediatrics, Division of Pediatric Pulmonology, Allergology and Endocrinology, Comprehensive Center Pediatrics, Medical University of Vienna, Vienna, Austria,; 12 Department of Dermatology, Venereology and Allergology, Leipzig University Medical Center,; 13 Department of Dermatology and Allergy, Hannover Medical School, and; 14 Comprehensive Allergy Centre Charité, Department of Dermatology, Venereology and Allergology, Charité-Universitätsmedizin Berlin, Germany

**Keywords:** adverse reaction to food, histamine intolerance, diamine oxidase, restrictive diet, diet modification

## Abstract

Adverse reactions to food or food ingredients are more often perceived than objectively verifiable. However, reliable laboratory tests are often lacking. As a result, people with perceived adverse reactions to food often follow extensive elimination diets for years and unnecessarily restrict their diet, as in the case of the frequently suspected histamine intolerance. In this condition, laboratory parameters such as the determination of diamine oxidase in serum have been shown to be inconclusive. The lack of symptom reproducibility calls into question the clinical picture of adverse reactions to ingested histamine. In order to approach persons with perceived histamine intolerance and to support them in moving from blanket restrictions, which are often unnecessarily strict, to effective personalized therapeutic strategies, the present guideline of the Working Group on Food Allergy of the German Society of Allergology and Clinical Immunology (DGAKI) in cooperation with the Medical Association of German Allergists (AeDA), the Pediatric Allergology and Environmental Medicine (GPA) as well as the Swiss Society of Allergology and Immunology (SGAI) and the Austrian Society of Allergology and Immunology (ÖGAI) recommends a practicable diagnostic and therapeutic approach.

## Background and aim of the guideline 

Histamine in food is often suspected as a trigger for a variety of health problems, however, scientific evidence for a causal relationship between ingested histamine and reproducible symptoms is limited and contradictory. For a long time, the condition was postulated to affect only adults and, in particular, patients with chronic spontaneous urticaria and other skin symptoms [1, 2, 3, 4, 5, 6, 7, 8,9 ]. Lately, gastrointestinal symptoms have been increasingly observed [10, 11, 12], and also reports of affected children are available [13, 14]. Merely the suspicion of histamine intolerance often leads to extensive dietary restrictions that affect not only diversity of diet but also social life. For fear of reactions and uncertainty about the presence of histamine, affected persons often restrict themselves much more than is reasonable from their medical history. 

This contrasts with an extremely limited body of evidence on the effect of low-histamine diets: The few studies that have been conducted are observational (three of them on chronic spontaneous urticaria), and do not allow causal conclusions to be drawn [6, 8, 9, 15, 16, 17, 18, 19, 20]. Randomized controlled trials are lacking. Consequently, the authors of a systematic review on the effect of diets in chronic spontaneous urticaria conclude that individual patients might benefit from diets, including the low-histamine diet, but the evidence for this is low [21]. Very few studies have performed double-blind, placebo-controlled oral food challenges with histamine and results have been quite heterogeneous [7, 18, 22]. 

***Ingested histamine is often suspected as the cause of (unspecific) health complaints despite the fact that the scientific evidence supporting such a clinical picture is limited and contradictory.***

Although various parameters are postulated for diagnosis of adverse reactions to ingested histamine, there are still no reliable laboratory tests that can be used to confirm or reject the diagnosis. 

In the present guideline, the Working Group on Food Allergy of the German Society of Allergology and Clinical Immunology (DGAKI), in collaboration with the Medical Association of German Allergists (AeDA), the Society for Pediatric Allergology and Environmental Medicine (GPA), as well as the Swiss Society of Allergology and Immunology (SGAI) and the Austrian Society of Allergology and Immunology (ÖGAI), discuss presumed mechanisms, postulated diagnostic parameters, and the existence of adverse reactions to ingested histamine. Furthermore, a practicable diagnostic and therapeutic approach is proposed to prevent (allegedly) affected persons from suffering due to extensive elimination diets and unnecessary restrictions. 

## Occurrence, functions, and degradation pathways of histamine 

**Figure 1:**
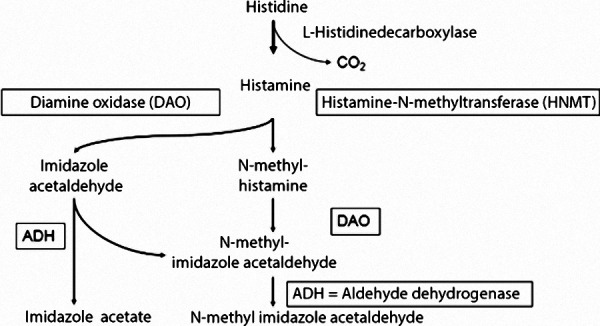
Degradation pathways of histamine.

Histamine is a biogenic amine. It is formed by endogenous synthesis from the amino acid histidine and is involved in many physiological processes. Its predominantly local effects are mediated by four receptors: Histamine H1 receptors which dilate blood vessels, constrict airways, and induce itching; H2 receptors which regulate gastric acid secretion; H3 receptors which regulate the sleep-wake rhythm; and H4 receptors which modulate the immune system. At the cellular and local level, histamine release and effects are carefully regulated – in part by histamine itself through its receptors [51]. Histamine formed endogenously and stored predominantly in mast cells and basophils is one of the major mediators of IgE (but also non-IgE) mediated clinical responses. Endogenous and exogenous histamine is eliminated via two degradation pathways (Figure 1):

- methylation by histamine N-methyltransferase (HNMT) and
- oxidative degradation by diamine oxidase (DAO).

HNMT is found intracellularly in most body tissues, whereas DAO is produced in the intestine, kidneys, and placenta and acts extracellularly. Using monoclonal antibodies, DAO has also been detected in seminal plasma samples but not in serum [23]. Histamine degradation may be impeded by simultaneous ingestion of alcohol because the same degradative enzymes are involved in degrading both [24]. 

Larger amounts of histamine can cause intoxication: Mild intoxications have been described for histamine amounts above 100 mg, and severe intoxications for amounts above 1,000 mg. The tolerated dose also depends on the matrix histamine is ingested. Histamine in cheese is tolerated much better than histamine in (spoiled) fish, especially fish from the Scombridae family (tuna, mackerel, etc.) [25]. The latter is a consequence of histamine overdose and not of intolerance to histamine, i.e., every human reacts to the intake of unnaturally large amounts of histamine. Nevertheless, there are doubts as to whether observed symptoms of fish poisoning are solely due to the histamine content of the spoiled fish [26]. 

## Diagnosis of adverse reactions to ingested histamine 

There is no reliable procedure to diagnose adverse reactions to ingested histamine. 

As diagnosis is mainly based on a patient’s medical history, after exclusion of other causes, it is reasonable to take a closer look at various relevant aspects in order to clarify the following questions: What symptoms can be expected and which differential diagnosis could be helpful? Is there evidence of the presumed mechanisms? Which diagnostic parameters have been described and how reliable are they? Can drug therapy influence the clinical picture? In addition, considerations about confirming a suspected diagnosis by oral food challenges and the difficulties that arise due to variable contents in foods are discussed. 

### Symptoms and differential diagnosis in patients with suspected adverse reactions to ingested histamine

Histamine receptors are located in many organs in the body accounting for a complex symptomatology if histamine is assumed to be the trigger. Sudden erythema on the face (flushing) but also itching and erythema on the body are reported as classic symptoms of adverse reactions to ingested histamine. Gastrointestinal symptoms such as nausea and/or vomiting or diarrhea and abdominal pain can also be triggered by histamine. Symptoms affecting the respiratory tract and cardiovascular symptoms, such as hypotension, dizziness, or tachycardia, are rarer but not unknown [2, 4, 27]. 

Since all the symptoms described could also be triggered by endogenously released histamine, a broad differential diagnosis may be necessary to identify other underlying pathomechanisms. These include skin diseases such as (chronic) urticaria, but also gastrointestinal diseases such as chronic inflammatory bowel diseases, carbohydrate metabolism disorders (e.g., lactose intolerance, fructose malabsorption), celiac disease as well as mastocytosis and allergic diseases (Table 1). 

### Presumed mechanism of action

Since the mid 1980s, ingested biogenic amines have been suspected of causing adverse reactions in some individuals even in small quantities below the toxic dose. The discussion has focused on adverse reactions elicited by histamine-containing foods, despite the fact that other biogenic amines (cadaverine, tryptamine, tyramine, serotonin, etc.) and/or polyamines (putrescine, spermine, spermidine, and others) may also cause adverse reactions or affect histamine metabolism, respectively [1]. The suspected mechanism of action for adverse reactions to ingested histamine involves impaired degradation by catabolizing enzymes, primarily DAO [4]. The term “histamine intolerance” was coined with reference to the term “lactose intolerance” (due to an enzyme deficiency). However, prospective, controlled studies reliably demonstrating that an enzyme and/or deficient enzyme activity is the cause of adverse reactions to ingested histamine are still lacking. Furthermore, it must be borne in mind that histamine is degraded not only by DAO but also by HNMT (Figure 1). 

***The causal link between adverse reactions to ingested histamine and an impaired histamine catabolism due to a DAO deficiency is still lacking. Therefore, we will exclusively use the term adverse reactions to ingested histamine.***

Two studies claiming that oral supplementation of DAO may improve symptoms in patients were supported by the manufacturer of the capsules [22, 28]. The first study aimed to “objectify and quantify histamine-associated symptoms and to analyze whether oral administration of the histamine-degrading enzyme DAO caused a reduction of symptoms” [22]. In 39 patients who had responded initially to an open challenge with 75 mg histamine in peppermint tea, neither major nor minor symptoms could be reproduced by double-blind, placebo-controlled challenge. Thus, the primary endpoint of the study was not met, and it is unclear how the authors concluded that intake of DAO capsules resulted in a “statistically significant reduction in symptoms”. 

The second study was only observational without a control group: In a pilot study symptomatology with and without DAO use was compared in 28 patients. The chosen design was not suitable to show causal effects, and carried a high risk of describing placebo effects [28]. 

***The effectiveness of DAO supplementation has not been scientifically proven to date and is not recommended.***

### Described diagnostic parameters with regard to their significance

Various parameters have been proposed for the diagnosis of “histamine intolerance”, their reliability will be discussed below. 

**DAO activity in serum**

Diagnosis based on measurement of DAO enzyme activity in the blood cannot be considered conclusive. Results of several studies suggest that the DAO values of affected and healthy individuals are comparable [29, 30]. Using DAO-specific monoclonal antibodies, it was not possible to detect relevant amounts of DAO in serum. However, this was possible in known tissues such as the kidney, intestine, and placenta, as well as in seminal plasma [23]. This observation strongly calls into question the significance of a measurement of DAO in serum. 

Nevertheless, recent studies repeatedly proclaim the significance of DAO measurement in serum or use it to identify affected individuals with “histamine intolerance” [11, 15, 16, 31]. 

***Measurement of DAO activity in serum has no diagnostic value.***

**Histamine 50 prick test**

A study that could support the suspicion of slowed histamine degradation in affected persons was published by Kofler et al. [32]. As part of the histamine 50 prick test, the wheal of the positive control (histamine wheal) is re-read after 50 minutes. If the wheal size remains unaltered up to that time, an impaired degradation is assumed. However, this method does not permit conclusions to be drawn on whether ingested histamine is also degraded at a slower rate. Follow-up studies by the authors or other working groups are not available to date. 

**Measurement of enzyme activities in the intestine**

Potential diagnostic significance is attributed to the measurement of enzyme activity (or activities) (DAO and possibly HNMT) in the intestinal mucosa, since this is considered the most important organ for the degradation of exogenous sources of histamine. However, according to current knowledge, human DAO activity in serum – in contrast to animals – does not permit any conclusions to be drawn on enzyme activity of DAO in the small intestine [33]. 

Further scientific investigation is needed to establish whether determination of DAO enzyme activity in the small intestinal mucosa yields information on capacity to degrade exogenous histamine. 

Kuefner et al. [34] demonstrated a statistically nonsignificant trend towards decreased DAO activity in the colonic mucosa of patients with food allergies. In contrast, HNMT activity was markedly decreased. In parallel, histamine levels in the intestinal mucosa were elevated. The authors see this diminished HNMT activity as the primary cause of the impaired histamine metabolism in the colon. However, the study did not investigate the effect of ingested histamine. 

The same working group was able to show that not only DAO activity, but also – and even to a greater extent – HNMT activity was diminished in the affected tissue of patients with colonic adenoma [35]. In these patients, the authors found slightly increased histamine concentrations in bowel tissue; however, this did not correlate with enzyme activity. They concluded the histamine in the colonic mucosa was more likely to be elevated due to increased release rather than reduced degradation. Again, the effect of exogenous histamine intake was not investigated. 

***Histamine in the intestine is degraded not only by DAO, but possibly also by HNMT.***

**Histamine in stool samples**

It is now known that some bacteria of the intestinal microbiota, in particular *lactobacilli*, can secrete large quantities of histamine. This casts doubt on the validity of high histamine levels in stool samples considered as pathological. O’Mahony and his working groups [36, 37, 38] were able to demonstrate in a mouse model that histamine present in the intestinal lumen – depending on which histamine receptor it binds to – can exert not only pro-inflammatory but also regulatory effects on the immune system. If the histamine present binds to the histamine H2 receptor, it has a more regulatory effect [36, 37, 38]. 

***The fact that histamine is a relevant metabolite of intestinal bacteria calls into question the validity of diagnostics stool analysis.***

**Histamine concentrations in plasma**

The significance of determining plasma histamine levels is scientifically controversial. Giera and coworkers performed histamine challenges in patients with suspected histamine intolerance and controls with 75 mg histamine and placebo [39]. The rise in plasma histamine following verum administration was minimal in the patients with presumed histamine intolerance, and did not differ from that after placebo; neither did it occur in patients who exhibited symptoms in response to provocation. In contrast, in the control group, there was a marked rise in plasma histamine following verum administration, albeit without accompanying symptoms. 

**Methylhistamine in urine**

The determination of the methylhistamine content in urine must be critically questioned, since methylhistamine levels depend not only on the histamine content, but also generally on the protein content of the diet. Methylhistamine levels rise on a high-protein, but low-histamine diet [40]. 

***To date, there are no objective parameters to support the presence of adverse reactions to ingested histamine.***

### Importance of medications

A number of drugs have been attributed a negative influence on histamine-degrading enzymes, primarily on DAO [41, 42]. Drugs such as acetylcysteine, metamizole, verapamil, metronidazole, and metoclopramide have been mentioned [43, 44]. According to a recent literature search, data from these older reports is inconsistent. Further research is needed to validate the effect of these and other drugs on histamine-degrading enzymes and to identify potential pharmacologic interactions with ingested histamine.

***The relevance of certain drugs with regard to the degradation capacity of DAO needs to be validated in further studies.***

### Oral challenge with histamine: between diagnostic threshold dose and unintentional intoxication

The appropriate method for the unequivocal diagnosis or exclusion of an adverse reaction is a titrated oral provocation, ideally performed in a double-blind, placebo-controlled manner with clinically defined parameters as endpoints. For routine use in practice, there is no established procedure for suspected adverse reactions to ingested histamine. A prerequisite for diagnostically meaningful oral provocation is the determination of a reasonable provocation dose. The ideal dose should fail to trigger any reactions in a sufficiently large collective of healthy persons while inducing the described symptoms in persons suspected to be intolerant. If unexpected systemic reactions occur that were not described in the medical history, the administered dose was too high. 

The dose of 75 mg histamine, which was chosen in most studies available to date, triggered symptoms in half of the healthy subjects in one study [27]. In another study in patients with atopic dermatitis (AD), systemic reactions occurred in seven patients and four control subjects after administration of a dose of 0.75 mg histamine dihydrochloride (1 mg histamine corresponds to 1.6 mg histamine dihydrochloride) per kilogram body weight (mg/kg BW). After administration of a dose of 1.5 mg/kg BW, 14 AD patients and 11 control subjects reacted [18]. 

The fact that, in both studies, provocation doses trigged reactions in healthy controls prompts the suspicion of subtoxic effects. It is therefore doubtful whether these doses are suitable for diagnosing “hypersensitive” subjects. 

Additionally, in a multicenter study from Austria, symptoms were not reproducible after administration of 75 mg histamine [22]: While 39 of 56 patients clearly responded to histamine in peppermint tea in open provocation, none of these reactions were reproducible in double-blind, placebo controlled challenges. Furthermore, placebo responses occurred, suggesting that patient expectancy has a relevant influence on the provocation outcome. 

**Notes for practice**

If a histamine challenge is considered after the procedure described in “Practical approach in everyday life” – despite all uncertainties – it is recommended to perform a titrated provocation with ascending doses of histamine hydrochloride at 2-hour intervals (for example, 0.5 mg/kg BW, 0.75 mg/kg BW to 1.0 mg/kg BW) to determine the individually tolerated dose. 

Titrated histamine provocation should be performed under medical supervision, since systemic reactions ranging from nausea or vomiting to temporary circulatory dysregulation may occur. These symptoms are generally transient and can be managed by the administration of antihistamines. 

It must be borne in mind that individual sensitivity can vary considerably and that numerous accompanying factors affect intestinal permeability, including the following:

- The use of acetylsalicylic acid, other non-steroidal anti-inflammatory drugs as well as other medications,
- various, but primarily inflammatory, intestinal disorders,
- concomitant consumption of alcohol,
- hormone status,
- probably also the individual composition of the intestinal microbiota and other factors.

***If a clinical response to histamine is observed during titrated provocation but not to placebo, the triggering dose of histamine may be administered again in a placebo-controlled manner to exclude a chance event.***

### Variable histamine content in foods

The histamine content in foods varies greatly depending on maturity, storage time, and processing. Consequently, histamine levels can differ considerably within the same food product. For example, the level of histamine content in Emmental cheese varies from < 0.1 to 2,000 mg/kg or in smoked mackerel from < 0.1 to 1,788 mg/kg [45]. Consequently, it is difficult to estimate the histamine content of individual meals. Based on observations that histamine is tolerated differently depending on the food matrix [25] and that provocations with orally administered histamine are not reproducible [22], it is questionable whether a quantitative classification of foods with respect to histamine content is reasonable at all. 

Some of the dietary recommendations described are not scientifically proven [47]. For example, some foods are forbidden that do not even contain histamine in relevant amounts (e.g., yeast), others are to be avoided because of “histamine liberators” (pharmacologically active substances that are supposed to cause histamine release). To date, there is no reliable evidence of the existence of “histamine liberators” in foods nor of their clinical significance in adverse reactions to food or food ingredients [46]. 

***An assessment of the tolerance of histamine-containing foods based on the level of their histamine content is not reasonable, since levels vary widely and there is doubt that histamine is the (sole) triggering factor.***

## Practical approach in everyday life 

**Figure 2:**
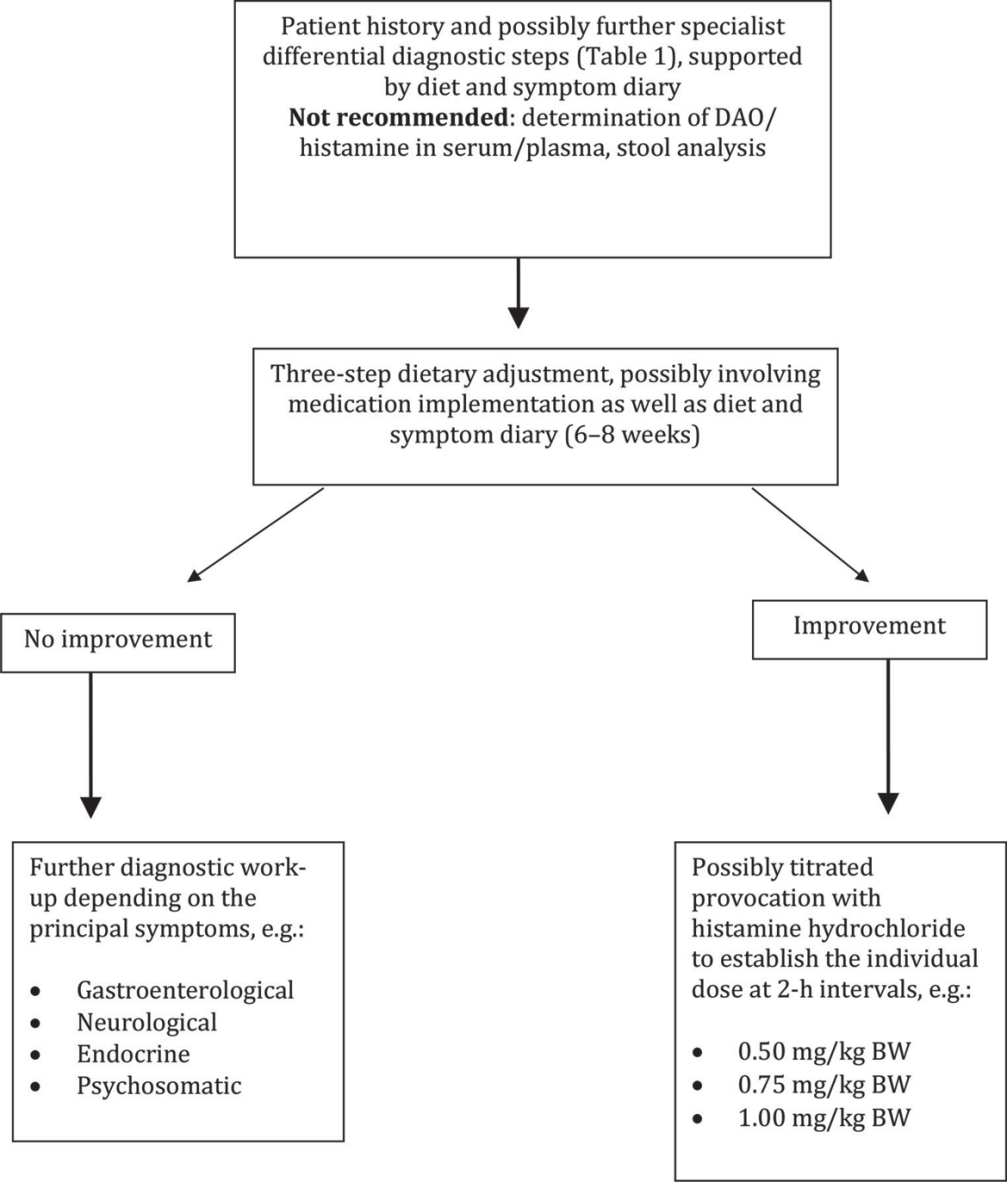
Proposed approach in patients with suspected adverse reactions to ingested histamine.

The following procedure is suggested for objectifying the occurrence of symptoms after histamine ingestion (Figure 2): 

As a first step, possible differential diagnoses (see “Symptoms and differential diagnosis in patients with suspected adverse reactions to ingested histamine”) should be investigated and, where necessary, treated. If the suspicion of an adverse reaction to ingested histamine persists, a symptom and food diary can be used to individually narrow down suspicious amounts of biogenic amines and to identify accompanying circumstances that induce or promote hypersensitivity reactions. Relevant factors that may increase sensitivity to histamine can have various causes and have already been addressed in “Oral challenge with histamine: between diagnostic threshold dose and unintentional intoxication”. In female patients, increased complaints are observed premenstrually [4]. 

The focus of the proposed approach is a three-step dietary adjustment (Table 2). This should not only be used to confirm or reject the suspected diagnosis, but also offers a return to an unrestricted diet or a personalized elimination. Affected individuals can benefit from a change in dietary behavior in favor of optimizing digestive functions. By changing the meal structure and combination of macronutrient proportions (fat, carbohydrates, and proteins) on the basis of a vegetable-based diet, gastric retention as well as transit times can be improved, thus creating optimized conditions for complete nutrient absorption. A similar approach has been established in the management of carbohydrate metabolism disorders, such as lactose intolerance or fructose malabsorption, and creates the basis for significantly fewer restrictions due to the improved digestive situation [47]. 

### Change of diet

Experience to date in the context of personalized nutritional therapies has shown that the tolerance of histamine and biogenic amines can be increased by a three-stage dietary change (Table 2). However, controlled studies are needed to investigate to what extent nutritional changes are able to achieve biological effects and influence the natural course of tolerance. Moreover, these studies could provide information on additional psychological effects occurring through expert advice. Despite the limited and contradictory knowledge about the underlying pathomechanism of adverse reactions to ingested histamine, a three-step dietary adjustment is recommended in order to approach (supposedly) affected individuals and to support them in moving from blanket and thus often unnecessary restrictions to effective personalized therapeutic strategies. 

***The approach of a diagnostic work-up, combined with personalized symptom-oriented nutritional therapy, which focuses primarily on nutrient optimization and leads patients reliably to differentiate symptoms, is recommended and to be preferred over generalized, restrictive diets.***

### Importance of antihistamines

There are no double-blind, placebo-controlled, prospective studies on the efficacy of H1 and H2 receptor blockers in patients with adverse reactions to ingested histamine. However, the mode of action of these drugs suggests that they ought to work in the treatment of individual symptoms (e.g., H1 blockers for flushing or H2 blockers for nausea/vomiting) – particularly in the acute setting (cumulative ingestion of foods with a high histamine content, for example, in the context of festive meals or scrombroid poisoning) [48, 49, 50]. 

In a pragmatic approach, one could conceivably treat patients with suspected adverse reactions to ingested histamine with H1/H2 receptor blockers for a certain period of time in order to investigate whether this alters symptoms. 

## Conclusion and outlook 

Diagnosis, but also rejection of adverse reactions to ingested histamine has to be made purely on the basis of symptoms. The proposed three-step dietary adjustment can be offered to (allegedly) affected persons if they restrict their diet considerably due to the perception of adverse reactions to ingested histamine and suffer greatly from an impaired quality of life, especially in the social context. 

Further research is needed to elucidate the relevance of biomarkers, factors impacting on gut function and barrier, as well as the histamine dose that elicits pharmacological effects. It should be taken into account that an important characteristic of histamine is its physiologically localized effect with careful regulation by itself. Thus, histamine and other vasoactive substances belong to the “autacoids”, i.e., vasoactive neurotransmitters that exert their effect in the immediate vicinity of their production site and are also degraded and down-regulated there. 

With the current state of knowledge, the primary goal is to prevent patients, by means of expert nutritional counseling, from following diets that lead to unnecessary dietary restrictions and thereby reduction in quality of life. 

## Consensus procedure 

A representative and multi-disciplinary expert group of the professional society or societies develops a recommendation by informal consensus, which is adopted by the board of the professional society or societies. The recommendations of the guideline were prepared by informal consensus of all experts after an extensive literature search. 

## Manager 

Prof. Dr. Margitta Worm, Berlin. 

## Funding 

None. 

## Conflict of interest 

The authors declare no conflict of interest. 

**Table 1.** Symptoms and differential diagnoses in patients with suspected histamine intolerance.

**Symptoms**	**Differential diagnosis/diagnoses**
Flush*	Neuroendocrine tumors; mastocytosis
Itching*	Urticaria, pruritus sine materia, prurigo
Nausea/vomiting*	Ventricular ulcer, duodenal ulcer
Diarrhea and abdominal pain*	Chronic inflammatory bowel diseases, carbohydrate utilization disorders (lactose intolerance, fructose malabsorption), celiac disease, matocytosis
Rhinitis*	Allergic and non-allergic rhinitis
Dyspnea, voice disorder*	Allergic and non-allergic asthma
Drop in blood pressure, dizziness, tachycardia*	Anaphylaxis, mastocytosis

*The analysis of symptoms in terms of temporal occurrence gives important differential diagnostic indications; only if there is a temporal association with food intake (minutes to 4 hours) is there a suspicion of food intolerances.

**Table 2.** Phases of the three-step change of diet.

**Phase**	**Aim**	**Recommendation**	**Duration**
1^st^ phase: Restriction phase with optimization of digestive conditions	Reduction of complaints to the greatest possible extent	Vegetable-based mixed diet with restriction of biogenic amine intake, especially histamine intake. The major dietary measure is the optimization of digestive conditions through: Nutrient optimization, Change of meal composition Principles of an adapted balanced diet (light whole food diet).	10 – 14 days
Phase 2: Test phase	Expansion of food selection taking into account individual influencing factors (stress, menstruation, medication intake, etc.)	Targeted reintroduction of suspected foods while maintaining optimized digestive functions. Soften dietary restrictions. Determination of individual histamine tolerance.	Up to 6 weeks
3^rd^ phase: Permanent nutrition	Permanent, demand-covering nutrient supply with high quality of life	Individual dietary recommendations based on optimized digestive functions, based on the individual’s histamine tolerance taking into account exogenous influencing factors.
